# Bacterial community diversity and variation in spray water sources and the tomato fruit surface

**DOI:** 10.1186/1471-2180-11-81

**Published:** 2011-04-21

**Authors:** Adriana Telias, James R White, Donna M Pahl, Andrea R Ottesen, Christopher S Walsh

**Affiliations:** 1 Plant Science and Landscape Architecture Department, University of Maryland 2102 Plant Sciences Building, College Park, MD 21201, USA; 2 Institute for Genome Sciences, University of Maryland School of Medicine 801 West Baltimore St., Baltimore, MD 21201, USA; 3 Division of Microbiology, Center for Food Safety and Applied Nutrition, US Food and Drug Administration, 5100 Paint Branch Parkway, College Park, MD 20740, USA

## Abstract

**Background:**

Tomato (*Solanum lycopersicum*) consumption has been one of the most common causes of produce-associated salmonellosis in the United States. Contamination may originate from animal waste, insects, soil or water. Current guidelines for fresh tomato production recommend the use of potable water for applications coming in direct contact with the fruit, but due to high demand, water from other sources is frequently used. We sought to describe the overall bacterial diversity on the surface of tomato fruit and the effect of two different water sources (ground and surface water) when used for direct crop applications by generating a 454-pyrosequencing 16S rRNA dataset of these different environments. This study represents the first in depth characterization of bacterial communities in the tomato fruit surface and the water sources commonly used in commercial vegetable production.

**Results:**

The two water sources tested had a significantly different bacterial composition. Proteobacteria was predominant in groundwater samples, whereas in the significantly more diverse surface water, abundant phyla also included Firmicutes, Actinobacteria and Verrucomicrobia. The fruit surface bacterial communities on tomatoes sprayed with both water sources could not be differentiated using various statistical methods. Both fruit surface environments had a high representation of Gammaproteobacteria, and within this class the genera *Pantoea *and *Enterobacter *were the most abundant.

**Conclusions:**

Despite the major differences observed in the bacterial composition of ground and surface water, the season long use of these very different water sources did not have a significant impact on the bacterial composition of the tomato fruit surface. This study has provided the first next-generation sequencing database describing the bacterial communities living in the fruit surface of a tomato crop under two different spray water regimes, and therefore represents an important step forward towards the development of science-based metrics for Good Agricultural Practices.

## Background

An increasing number of epidemic outbreaks caused by contamination of produce by human pathogens have been observed in the United States [1]. Between 1996 and 2008, a total of 82 produce related outbreaks were reported. Bacterial species comprise the majority of reported disease causing agents, with pathogenic *Salmonella *and *E. coli *strains implicated most frequently. Lettuce and tomatoes were the commodities associated with the most outbreaks, followed by cantaloupe and berries [2]. In recent years, tomatoes have been one of the main products responsible for produce-associated salmonellosis [3].

The phyllosphere has found itself at an intersection of food safety concerns and research that examines the microbial ecology of agricultural environments [4-6]. Human pathogens find their way to this environment via diverse channels that remain poorly understood. Human, animal, atmospheric, abiotic and xenobiotic conduits have all been examined for their potential to contribute to the precise factors needed to support growth or simple persistence of human pathogens of bacterial origin in agricultural commodities [7,8]. An extremely important component of agricultural management that remains to be comprehensively examined with culture-independent methods is the microbial ecology associated with water sources used in irrigation and pesticide applications.

In the United States, the tomato industry's Good Agricultural Practices guidelines, which are focused on improving the food safety of the product, recommend the use of potable water for applications that come in direct contact with the crop [9]. Given that large volumes of water are needed for pesticide applications and overhead irrigation of vegetable crops, water demand cannot always be met with the available potable water. Consequently growers routinely use water from other sources, such as farm ponds. Surface water is highly susceptible to contamination due to direct discharge of sewage and the impact of runoff. In the mid-Atlantic region of the United States growers report routine visits to their farm ponds by Canada geese, a potential avian reservoir of *Salmonella *[10] and white-tailed deer, a potential reservoir for *E. coli *O157:H7 [11]. This region is home to a large poultry industry, which also represents a potential source of *Salmonella *contamination. Groundwater sources, on the other hand, are less likely to support enteric pathogens because of the natural filtering mechanisms of soils, although poorly managed wells are susceptible to contamination [12].

The type of irrigation system can influence the risk of crop contamination: overhead irrigation, for instance, is more likely to produce virus contamination than are furrow and drip irrigation [13]. Studies conducted in California found no significant differences in coliform counts among crops spray-irrigated with two types of treated wastewater or with well water. This was found despite the fact that the treated waters used in this study showed higher levels of total and fecal coliforms than the well water [14]. The overall impact of using surface water for direct crop applications on fruit surface bacterial communities has not been reported to date.

Denaturing gradient gel electrophoresis studies have indicated that variables such as plant species and stage of development can affect the composition of phyllosphere microbial communities. In addition, it was found that these communities are far more complex than culture-based methods used in the past had indicated [6,15,16]. Recent studies described the bacterial diversity of phyllosphere samples from natural and agricultural ecosystems using traditional cloning and sequencing approaches, leading to the identification of many previously undescribed members of these communities. These studies also indicated that phyllosphere communities can be altered by the application of diverse agricultural materials [16-18].

More recently next-generation sequencing technologies, including 454-pyrosequencing, have provided more comprehensive descriptions of bacterial communities in different environments due to the increased number of sequence reads obtained [19-26]. A study of bacterial diversity on tree leaves using 454 sequencing indicated that tree and bacterial community phylogeny are associated, and that the geographic differentiation of bacterial communities on a single tree species is minimal [27]. To our knowledge, no such studies have been conducted to date to describe the impact of water quality on bacterial populations in the phyllosphere of specialty crops.

We utilized 454-pyrosequencing to generate 34,016 16S rRNA gene sequences from 16 field samples: 10 tomato fruit samples that had been sprayed with either surface water (*ps*), or groundwater (*pg*), three samples of surface water (*ws*), and three samples of groundwater (*wg*). Using these data, we sought to 1) compare the bacterial profile of ground and surface water that was used for pesticide applications and 2) assess the impact of water quality on the fruit surface bacterial profile of a tomato crop. A smaller preliminary dataset of 2008 fruit surface samples generated through Sanger sequencing is also included for comparison. Despite the significant differences between bacterial communities in surface and groundwater, the surface communities on the tomato fruits treated with these water sources could not be differentiated by a variety of statistical methods.

## Results

### Taxonomic distributions among samples

After screening our data for poor sequences and contaminants (see Methods), we recovered 27,757 high-quality 16S rRNA gene sequences with an average of 1,734 ± 471 (SD) sequences per sample (results refer to the 2009 data unless otherwise stated).

**Figure 1:**
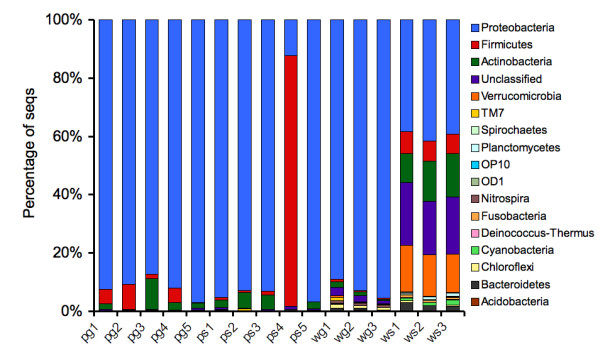
**Phylum level abundance profiles using 16S rRNA sequence classifications**. Columns reflect the percentage of 16S rRNA sequences assigned to each phylum using the RDP classifier. All *ws *samples show a more even representation of Proteobacteria, Firmicutes, Actinobacteria, and Verrucomicrobia, as well as a higher representation of sequences that could not be assigned to any phylum (with a confidence threshold of 80%). We also observe a spike in Firmicutes abundance in a surface water-treated phyllosphere sample 4 (*ps4*). In all other samples, Proteobacteria 16S rRNA sequences tend to dominate the profiles.

We taxonomically classified all sequences (from phylum to genus) using the RDP Bayesian classifier with a confidence threshold of 80%. Examining the phylum level distributions across samples, we found that nearly all fruit surface samples appeared to have very similar 16S rRNA profiles. In these, Proteobacteria dominated the observed sequences, with smaller representations of Firmicutes and Actinobacteria. One surface water treated sample (*ps4*) was dominated by Firmicutes sequences, most likely as a result of contamination with internal fruit material. While the *wg *samples displayed similar 16S rRNA profiles dominated by Proteobacteria, the *ws *samples had a more even representation among four dominant phyla. In addition, *ws *samples contained a large number of sequences that could not be classified even at the phylum level (Figure 1).

To compare environments for differentially-abundant taxonomic groups, we ran the Metastats methodology [28] on phylum, class, and genus level assignments. However, a limitation of the Metastats approach for q-value (individual false discovery rate) estimation is poor accuracy for datasets with < 100 features. To compensate, we compute the overall false discovery rate (FDR) for taxonomic groups we have called significant in our analysis using the method by Benjamini and Hochberg [29].

Results of Metastats runs comparing bacterial classes among populations and accounting for intra-replicate variability indicated that five taxonomic classes are differentially abundant in the two water sources (P < 0.015), most notably Betaproteobacteria, which makes up approximately 86% of sequences on average in the *wg *samples, but only close to 9% of sequences in the *ws *samples (Additional file 1). Of the five taxonomic classes we call as differentially abundant between *wg *and *ws *samples, the FDR ~0.12, so we expect less than one false positive among these five. The most abundant classes in *ws *profiles were Alphaproteobacteria, Actinobacteria and the unclassified group.

Betaproteobacteria was also the most differentially abundant class when *pg *and *wg *were compared (10 vs. 86%), among nine differentially abundant bacterial classes (FDR ~0.07). Fourteen bacterial classes were differentially abundant between *ws *and *ps *(FDR ~0.06), most notably Clostridia, which was enriched for in *ws*. Both fruit surface environments were enriched for Gammaproteobacteria. Despite the differences observed between water sources, no significant differences were found between the two fruit surface environments (this includes an attempt in which the *ps4 *outlier was removed).

At the genus level, significant differences were found between water sources, with 30 genera showing differential abundance (P < 0.05). Table 1 lists the bacterial genera among these representing 1% or more of the sequences in either of the water sources analyzed. Fruit surface environments were highly variable and no significant differences were detected for the high abundance genera, which included *Pantoea*, *Enterobacter, Sphingomonas*, *Leuconostoc, Pseudomonas *and *Burkholderia *(Additional file 2). The less abundant genera *Paenibacillus*, *Stenotrophomonas*, *Bacillus *and *Lactococcus *were more abundant in *pg*, while *Frigoribacterium*, *Herbaspirillum*, *Rickettsia*, *Wautersiella *and *Cloacibacterium *were more abundant in *ps*. None of these genera represented more than 0.2% of the population.

**Table 1:** Bacterial genera with differential abundance in ground and surface water sources.

Genus	Groundwater		Surface water		p-value
	**Mean**	**St. error**	**Mean**	**St. error**	

Acidovorax	0.018	0.005	0.001	0.001	0.039

Burkholderia	0.744	0.046	0.001	0.000	0.001

Clostridium	0.001	0.001	0.014	0.003	0.024

GpIIa	0.000	0.000	0.011	0.002	0.017

Ilumatobacter	0.000	0.000	0.011	0.003	0.025

Methylocystis	0.009	0.002	0.082	0.007	0.007

Mycobacterium	0.001	0.000	0.032	0.008	0.035

Polynucleobacter	0.000	0.000	0.016	0.001	0.008

Ralstonia	0.016	0.003	0.000	0.000	0.021

Spartobacteria_genera_incertae_sedis	0.000	0.000	0.078	0.009	0.010

Unclassified	0.110	0.021	0.684	0.019	0.000

Average relative abundance of sequences assigned to that genus (Mean), standard error of the corresponding average (St. error) and p-value describing the significance of the differential abundance observed between the two populations, for genera representing at least 1.0% of the sequences in either of the water sources. The computed FDR of these genera is 0.05, thus we expect that less than 1 of the 11 represent false positives.

**Figure 2:**
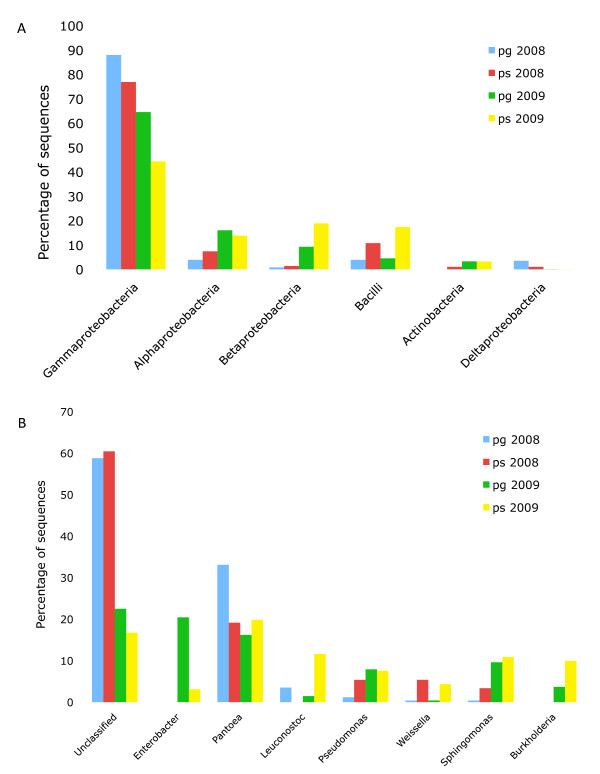
**Most abundant bacterial classes and genera in tomato fruit surface samples (2008 and 2009)**. A) Bacterial classes in surface and groundwater treated fruit surfaces, indicating a predominance of Gammaproteobacteria in both years. B) Bacterial genera in surface and groundwater treated fruit surfaces.

A statistical comparison of the 2008 and 2009 fruit surface samples (not considering variability between 2009 replicates) indicated that in both the 454 and Sanger data, Bacilli is enriched in the *ps *samples, and Gammaproteobacteria is enriched in *pg *(Figure 2A). At the genus level, *Pantoea *showed high abundance in both years (Figure 2B). *Enterobacter*, *Pseudomonas*, *Sphingomonas *and *Burkholderia *were more predominant in the 2009 samples, while a larger proportion of the 2008 sequences remained unclassified. These results indicate that we were able to detect similar bacterial populations on the tomato fruit surface in both years, despite the methodological differences, the differences between growing seasons and the fact that different tomato cultivars were sampled.

### Diversity analysis using operational taxonomic units

**Figure 3:**
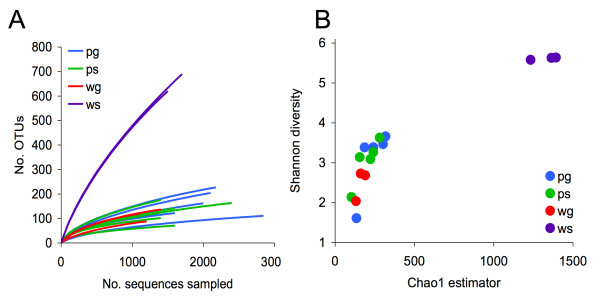
**OTU-based bacterial diversity analysis of water and crop samples**. (A) Rarefaction curves displaying the average number of OTUs discovered by random sampling within each sample. We observe a higher diversity in all surface water samples (*ws*) relative to fruit surface and groundwater samples. (B) This increased diversity is also apparent through the Chao1 and Shannon diversity estimators. To avoid bias due to different sampling depths, we first rarefied the data by randomly selecting 1100 sequences from each sample. Note that Chao1 estimates for total species-level diversity in surface water samples consistently exceed 1000 species, while all other environments fall below 500.

To compute estimates of species-level diversity and perform comparisons between environments, all sequences were clustered into operational taxonomic units (OTUs) using Mothur [30] and a similarity threshold of 95% (see Methods). The total number of unique OTUs within each environment was 494 (*pg*), 399 (*ps*), 228 (*wg*) and 1342 (*ws*). After computing rarefaction curves for each sample (Figure 3A), we immediately observed that the surface water samples were significantly more diverse than the others, and that groundwater and fruit surface samples are indistinguishable in terms of diversity. Additionally, the Shannon diversity index and Chao1 estimator were calculated for each sample, and again we see that the *ws *samples are the most diverse at the OTU level (Figure 3B).

To assess the diversity captured with the samples, we calculated the *Good's Coverage Estimator *on the OTUs from each sample using Mothur. Results indicated that we captured between 93 and 98% of the species in all of the samples except for *ws *samples, where we only identified between 70 and 73% of the species.

We then examined shared OTUs between individual replicates and treatments. Fruit surface environments shared approximately half their OTUs, and these represented more than 90% of the sequences in both samples. In contrast, water environments shared only 31 OTUs, which represented 2% of the OTUs present in surface water and 14% of those in groundwater. These shared OTUs corresponded to 62% of the sequences in groundwater, but only 6% of the sequences in surface water. These results again point to the greater differences between water-based microbial communities as compared to those in the treated tomato fruit surfaces.

**Figure 4:**
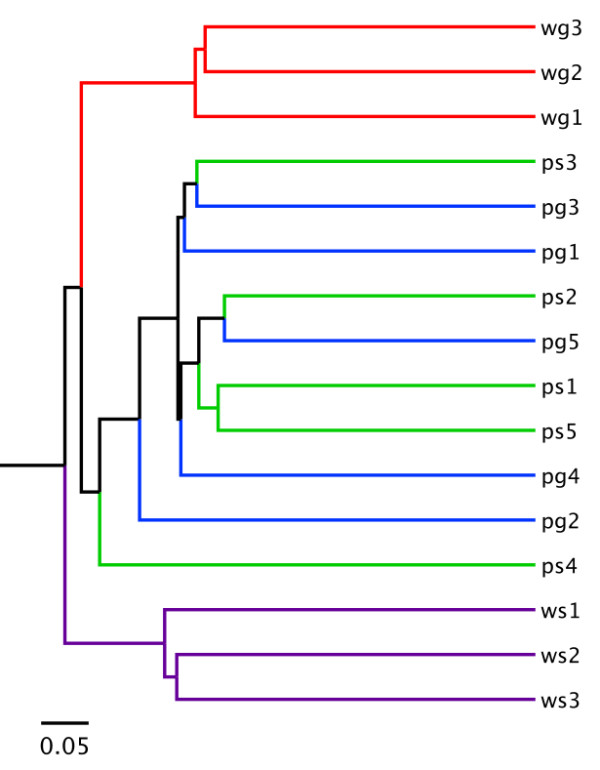
**Hierarchical clustering of samples using the Jaccard index**. Using shared OTU profiles across all samples, we computed Jaccard indices for clustering samples based on overall community similarity. Samples from each water environment cluster well, but even using OTU resolution, the fruit surface samples were not easily distinguishable.

A hierarchical clustering of all samples was performed using the Jaccard index based on shared OTU composition (Figure 4). This tree indicated that the two fruit surface communities are not uniquely distinguishable at the OTU level despite the microbial differences in water sources. However, water samples did cluster with their associated environments.

### Alternative methodologies

To test the sensitivity of the above results to any particular methodology, we re-ran our analysis using the new automated 16S rRNA pipelines provided by the CloVR software package (http://clovr.org). CloVR is a virtual machine designed to run large-scale genomic analyses in a cloud-based environment such as Amazon EC2. The CloVR-16S track runs Mothur [30] and Qiime-based [31] standard operating protocols in parallel complete with alpha and beta diversity analysis of multiple samples.

After running our high-quality sequence dataset through the CloVR-16S pipeline, we saw remarkable consistency with our initial results. All OTU analyses confirm the enriched diversity of surface water samples as compared to all others, as well as a lack of differentially abundant taxonomic groups between *pg *and *ps *samples.

**Figure 5:**
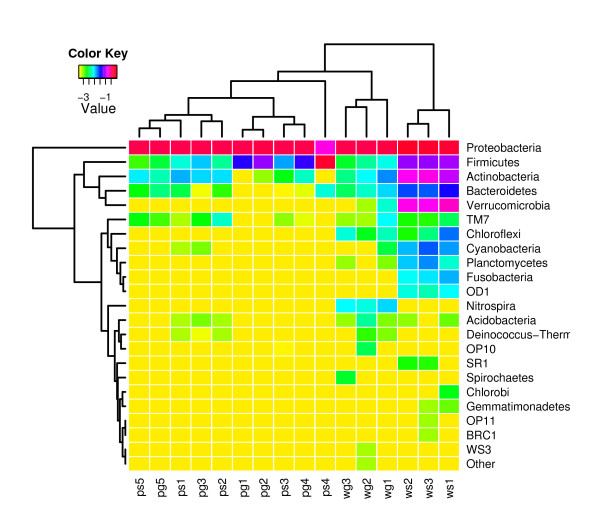
**Hierarchical clustering of samples using phylum level distributions**. Employing an alternative Qiime-based methodology to analyze our sequences, we see that water samples consistently cluster within their own specific environments. Again, this is not so for the fruit surface samples. Displayed values are log transformed relative abundances within each sample, (e.g. 0.10 ~-1; 0.01 ~-2). Visualized using skiff in CloVR.

**Figure 6:**
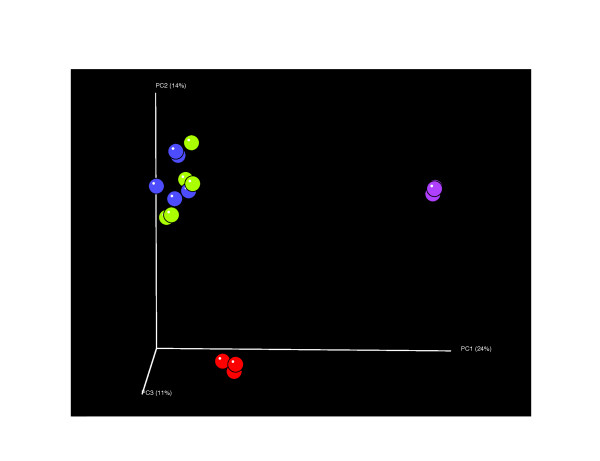
**Community analysis using principal coordinate analysis (PCoA) of unweighted UniFrac distance matrix**. Across all methodologies assessed, (including the canonical UniFrac beta-diversity analysis), water samples cluster very well, yet the phyllosphere treatments are unable to be differentiated. Displayed color scheme: *ps *(green), *pg *(blue), *ws *(purple), *wg *(red). Percentage of variation explained by each principal coordinate is shown on respective axes.

Using various unsupervised approaches, water samples consistently clustered with their unique environments at all taxonomic levels (Figure 5). There was persistent difficulty distinguishing between fruit surface samples treated with surface or groundwater. Even the UniFrac metric, which arguably maintains the highest phylogenetic resolution of any method, was unable to resolve this issue (Figure 6). The concordance among our methodology and the CloVR-16S methods suggests that our results are not sensitive to modifications in the analysis protocol.

### Screening for Enterobacteriaceae pathogens

Less than 1% of the hits in the water samples were to the family Enterobacteriaceae (Table 2). In fruit surface samples 33 to 79% of the sequences were identified as Enterobacteriaceae, with higher counts in *pg *than in *ps *in 2008 and again in 2009. Among the Enterobacteriaceae genera, *Pantoea *was the most abundant in both years. *Enterobacter *also showed high abundance, but only in the 2009 samples.

**Table 2:** Distribution of the Enterobacteriaceae family.

	*pg *2008	*ps *2008	*pg *2009	*ps *2009	*wg *2009	*ws *2009
Total sequences assigned to anything	257	298	10849	8567	3805	4536

Total RDP hits to Enterobacteriaceae	202 (78.6)	151 (50.7)	5716 (52.7)	2900 (33.9)	15 (0.39)	1 (0.02)

BLASTN total hit counts	198	147	5025	2760	14	1

BLASTN hits to Pantoea species	172 (86.9)	91 (61.9)	1191 (23.7)	1546 (56.0)	1 (7.14)	0

BLASTN hits to Enterobacter species	2 (1.01)	35 (23.8)	1665 (33.13)	567 (20.5)	7 (50.0)	0

BLASTN hits to Citrobacter species	0	0	3 (0.06)	1 (0.04)	0	0

BLASTN hits to Tatumella species	0	0	208 (4.14)	0	0	0

BLASTN hits to Cronobacter species	0	0	49 (0.98)	25 (0.91)	0	0

BLASTN hits to Erwinia species	0	2 (1.36)	7 (0.14)	4 (0.14)	0	0

BLASTN hits to Escherichia species	2 (1.01)	5 (3.40)	52 (1.03)	3 (0.11)	0	0

BLASTN hits to Klebsiella species	0	2 (1.36)	8 (0.16)	3 (0.11)	0	0

BLASTN hits to Trabulsiella odontotermitis	0	0	3 (0.06)	8 (0.29)	0	0

Hits to other Enterobacteriaceae	22 (11.11)	12 (8.16)	1839 (36.6)	603 (21.9)	6 (42.9)	1 (100)

Number of RDP hits to Enterobacteriaceae in tomato fruit surfaces and water samples and BLASTN hits to the different genera within the family (percentages are indicated between parentheses).

**Figure 7:**
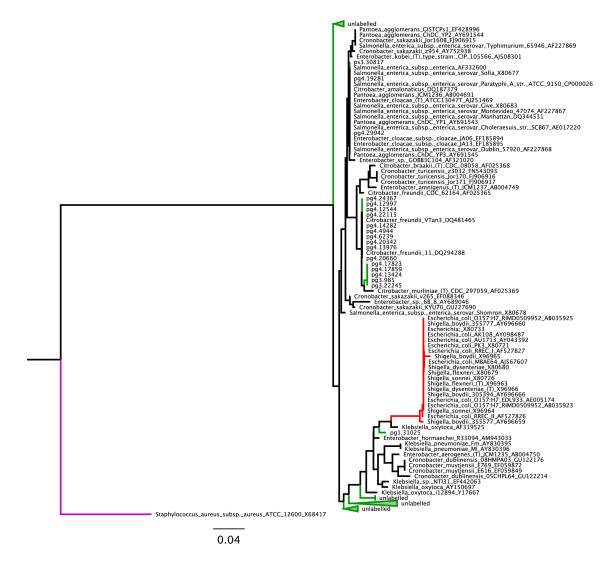
**Neighbor-joining phylogenetic tree of reads mapping to members of the Enterobacteriaceae family**. Screening our dataset for putative *E. coli*/*Shigella*/*Salmonella *species we discovered most hits were from the fruit surface samples. We found that by including 16S rRNA reference sequences from members of other related genera including *Citrobacter *and *Cronobacter*, we could not confidently assign any sequences from our dataset to *Salmonella *due to poor phylogenetic resolution. However, we did determine that no reads mapping to the Enterobacteriaceae family were from *E. coli*/*Shigella*. The *E. coli*/*Shigella *monophyletic clade is colored in red, the *Staphylococcus *aureus outgroup is purple, and monophyletic clades of sequences from our dataset are colored in green.

We created a phylogenetic tree in order to compare the Enterobacteriaceae species present in the different samples (Figure 7). By populating the tree with several genera we could not confidently assign sequences to pathogenic species within the family. Based on our tree, the 527 bp segment of the 16S rRNA gene used is not enough to distinguish between several members of the Enterobacteriaceae family.

## Discussion

This study provides the first next-generation sequencing survey of the bacterial community in the tomato fruit surface. As such it has confirmed the presence of taxa previously found to inhabit the phyllosphere of this crop species, as well as identified many others not yet encountered in this environment. The three most abundant bacterial classes in the tomato fruit surface environments compared in this study were Gamma, Alpha and Betaproteobacteria. These were also found in higher abundance in the phyllosphere of other plant species, although the relative abundances for these classes vary [16-18,27]. Genera here found in high abundance in the tomato fruit surface, such as *Pantoea *and *Enterobacter*, are also abundant in the phyllosphere of certain Atlantic rainforest tree species and cottonwood, indicating a wide distribution across different plant species [16,18]. Bacterial genera found in our 2009 fruit surface samples were also identified among the culturable bacteria on leaves of field-grown tomatoes, including *Pseudomonas*, *Pantoea*, *Sphingomonas*, *Massilia*, *Xhantomonas *and *Curtobacterium *[32]. Two additional genera, *Burkholderia *and *Leuconostoc*, showed high abundance in our study. *Burkholderia *was the most abundant genus in our groundwater samples, representing 75% of the sequences, and might have been introduced in the environment through groundwater applications. *Leuconostoc *has been previously described as the predominant lactic acid bacteria on tomato fruit surfaces [33].

Similar bacterial classes and genera were found in high abundance in samples collected in 2008 and 2009, with the largest differences corresponding to the unclassified sequences. Several different reasons could account for this variation, including differences in DNA extraction, sequencing sample preparation and primers used in both years, as well as potential growing season effects.

Of special interest is the high proportion of sequences identified as Enterobacteriaceae, given that this family includes important human pathogenic bacteria like *Salmonella *and *E. coli*. Similar representation of this family was obtained in the phyllosphere of *Trichilia *spp. and *Pinus ponderosa*, but not in that of *Campomanesia xanthocarpa *[16,27]. The high adaptability of this family to the tomato fruit surface environment might be associated to the higher risk of disease outbreaks associated with this crop.

Differences between fruit surface environments do not appear to be linked to the water applications, indicating that plant conditions allow for only some of the bacterial groups present in water to establish themselves. Similar results were obtained when the fruit surface communities living on apple trees under conventional and organic management were compared, where only low abundance groups differed between the two environments [17]. Similarly, no effect on the levels of fecal and total coliforms was observed when reclaimed water with higher coliform counts, and well water were sprayed on six horticultural crops [14].

Several factors determine whether the microorganisms arriving on the leaf surface can become established, including leaf characteristics, environmental factors and properties of the microorganisms themselves [8]. Pesticides are known to differentially impact bacterial survival and growth. In a study conducted to determine the effect of pesticides on bacterial survival, *Salmonella *spp. were best able to survive and *Listeria *spp. were least able to survive in pesticide solutions, among all the bacteria tested. Bravo, the fungicide applied closest to the sampling date in this study, has been found to reduce bacterial growth, although it was less inhibitory than other products tested [34]. The addition of pesticides to the different water sources used in this study might have reduced bacterial community differentiation in the two resulting fruit environments. The smooth texture of tomato skin may also prevent attachment and result in bacteria being washed away by rain or spray water.

Although our results point to the lack of major effects of the two water sources used for pesticide applications, confirming this at the species level for human enteric pathogens such as *Salmonella*, would be crucial for establishing the potential safety of surface water use for contact applications. In addition, our sampling depth analysis suggests that deeper sampling is needed for all the environments, but especially for the more diverse *ws*, to capture at least 90% of the community members

Recent studies of analysis methodologies in bacterial diversity and metagenomics projects have revealed that small modifications or substitution of similar tools may potentially result in significant changes in the overall biological conclusions [35-37]. In the rapidly evolving field of genomics, there are few concrete standards, and the sophisticated computational protocols being developed certainly will always be sensitive to some uncertainty in the analysis parameters. To examine the sensitivity of our results to the methodology employed, we re-ran our analysis using two parallel 16S rRNA protocols from the CloVR package and found large agreement with our major results. Additionally, the 454 platform itself has ongoing issues regarding artificial replicate generation [38] and homopolymer identification errors [39], both of which contribute to overestimation of species-level diversity in 16S rRNA-based studies. Though it is likely that our estimates of absolute species-level diversity are indeed inflated, the consistency in relative diversity differences between samples across multiple analyses is encouraging and lends support to the validity of our initial computational results and final biological and ecological conclusions.

## Conclusions

Our research has generated the first culture-independent next-generation sequencing data set for the bacterial microbiology associated with the phyllosphere of a tomato crop under agricultural management. There are a myriad of agricultural practices that may play a role in the contamination of tomatoes by human pathogenic bacteria. This work has provided valuable evidence suggesting that water used for pesticide applications does not represent a major modifier of the fruit surface bacterial communities composition.

As previously reported for other plant species, Gamma, Alpha and Betaproteobacteria and Bacilli comprised most of the 16S rRNA sequences identified in the tomato fruit surface, while the most abundant genera included *Pantoea*, *Enterobacter*, *Leuconostoc*, *Pseudomonas*, *Weissella*, *Sphingomonas *and *Burkolderia*. We suggest that the high representation of Enterobacteriaceae in the tomato fruit surface might be associated with the elevated food safety risks posed by this crop.

These results represent a major contribution to the understanding of the tomato fruit surface ecology and an important step towards the establishment of science-based metrics for Good Agricultural Practices that will ensure the safety of horticultural products. The emerging role of tomato as a model organism further emphasizes the value of a deeper understanding of the interactions between this crop species, its associated microflora and the environment.

## Methods

### Tomato crop

Field plots were established at the University of Maryland Wye Research and Education Center in Maryland's Eastern Shore (38°56', 76°07'). The soil was a Nassawango silt loam. Tomato transplants were planted in the field on June 9 2008 and June 10 2009. 'Sweet olive' (2008) and 'Juliet' (2009) grape tomato plants were planted on black plastic mulch and trained using stakes and a four-tier string system. The experimental design was a randomized complete block design with five blocks and three treatments. Seedlings were planted in paired rows (only one of them used for this study), 1.8 m apart. Each paired row was 9.0 m apart from the next set of paired rows. Within each row, each experimental unit was 9.0 m from the next. An experimental plot was composed of 3 grape tomato plants alternated with 2 'Brandywine' shipping tomato plants, which were not used for sampling (2008) or 5 grape tomato plants (2009) at an in-row spacing of 60 cm. In 2008, pesticides mixed in either ground or surface water were sprayed on: June 21, June 29, July 7, July 15, July 23, July 30, August 10 and August 30. In 2009, pesticides were sprayed on July 2, July 14, July 28, August 9, August 20 and August 30. Spray treatments were applied with a CO_2_-pressurized boom sprayer, using a separate sprayer manifold consisting of nozzles, hoses and a tank for each treatment. These booms were used throughout the season. Additional treatments (not used for this study) included organic managed plots (2008) and use of an additional pond as a source of surface water (2009). Standard agricultural practices for the production of shipping tomatoes in the region were used.

### Sample collection and processing

Samples consisting of 6 tomato fruits were aseptically collected on September 1 2008 and August 31 2009. Fruits were systematically harvested from different locations within the experimental unit and placed in Ziploc^® ^bags (2008) or Whirl-Pak^® ^bags (2009) by using new gloves for each replicate and ethanol disinfection of pruning shears between samples. Samples were then transported back to the laboratory at 4°C. One hundred milliliters of sterile water were added to the bags, and samples were agitated for 1 minute by hand and then sonicated for 2 minutes. The microfloral wash was then transferred to polypropylene tubes and centrifuged at 30,000 × g overnight at 4°C. The pellet was then transferred to a microcentrifuge tube and stored at -80°C until DNA extraction was performed. Three liters of groundwater and 50 ml of surface water collected on August 31 2009 were filtered through 0.45 μm Fisherbrand^® ^filters (Fisher Scientific, Pittsburgh, PA). Filters were aseptically divided into four microcentrifuge tubes and stored at 80°C. DNA extraction from filters and pellets was performed using the Promega Wizard DNA extraction kit (Promega, Madison, WI) in 2008, and the Zymo Research fungal/bacterial DNA extraction kit (Zymo Research, Orange, CA) in 2009.

### Cloning and Sanger sequencing (2008)

PCR amplification of the 16S rRNA bacterial gene was performed using forward primer GM5F 5'-CCTACGGGAGGCAGCAG-3' [40] and reverse primer 907R 5'-CCCCGTCAATTCCTTTGAGTTT-3' [41], designed to amplify a 588 base pair long region including the variable region V3. PCR reactions were performed using TaKaRa premix (TaKaRa Shuzo Co., Shiga, Japan) in a 50 μl total volume (1 μl genomic DNA as template, 1 μl each primer, 22 μl sterile water and 25 μl TaKaRa premix). PCRs used a denaturation step at 98°C for 5 minutes, followed by 30 cycles of 94°C for 1 minute, 55°C for 1 minute, 72°C for 1 minute, with a final extension step at 72°C for 5 minutes. PCR fragments were cloned into the pGEM^®^-T Easy Vector (Promega) according to manufacturer's instructions. Bacterial colonies were frozen in 100 μl aliquots of Luria broth (Miller) solution with 10% glycerol in 96-well plates and shipped on dry ice to Agencourt Genomic Services, Beverly, MA, for Sanger sequencing.

### 454 sequencing (2009)

PCR amplification of the 16S rRNA bacterial gene was performed using forward primer Bact-8F (AGAGTTTGATCCTGGCTCAG) [42] and reverse primer UNI518R (ATTACCGCGGCTGCTGG) [16], designed to amplify a 527 base pair long region including variable regions V1, V2 and V3. The forward primer included the fusion primer A (CGTATCGCCTCCCTCGCGCCATCAG) in its 5' end. The reverse primer included the fusion primer B (CTATGCGCCTTGCCAGCCCGCTCAG) in its 5' end, followed by sample specific 10 bp barcodes. Standard PCRs were performed using AmpliTaq Gold LD™ (Applied Biosystems, Foster City, CA) in a 50 μl total volume (1 μl genomic DNA as template, 1 μM each primer, 200 μM each dNTP, 2 mM MgCl_2_, 0.60 units AmpliTaq Gold LD, 10 × buffer provided by manufacturer). PCRs used a denaturation step at 95°C for 5 min, followed by 30 cycles of 95°C 1 min, 55°C 1 min, 72°C 1 min, with a final extension step at 72°C for 5 min. Four independent PCR amplifications were performed for each sample. After a gel based confirmation of PCR amplification, PCR products were purified using the AMPure kit (Invitrogen, Carlsbad, CA) following manufacturer's recommendations, and quantified using a Qubit flurometer (Invitrogen). PCR products were pooled and the average fragment size was assessed on a 2100 Bioanalyzer (Agilent, Santa Clara, CA) using a DNA 7500 chip. Emulsion-based clonal amplification and sequencing on the 454 Genome Sequencer FLX-Titanium system were performed at the W. M. Keck Center for Comparative and Functional Genomics at the University of Illinois at Urbana-Champaign according to the manufacturer's instructions (454 Life Sciences, Branford, CT). The PCR products were sequenced on two regions of a 16-region 70 × 75 picotiter plate. Signal processing and base calling were performed using the bundled 454 Data Analysis Software version 2.0.00.

### Initial sequence preprocessing

Recent validation studies have demonstrated several biases in analyses of 16S rRNA sequence datasets produced using 454-pyrosequencing technology [43]. We have deposited the 454 raw data in NCBI-SRA under the accession number SRX040888. To mitigate these issues for this study, 454 sequences were processed and analyzed using the following state-of-the-art procedures.

Sequences were first selected for length and quality according to the following criteria:

(i) ≥100 nucleotides in length (not including sample-specific barcodes)

(ii) a perfect match to a sample-specific barcode

(iii) reads were trimmed at the beginning of a poor quality region - defined as a 10 bp window containing 8 bp with a Phred-score ≤ 20.

Reads meeting the above criteria underwent rigorous screening for chimeric reads (using ChimeraSlayer (http://microbiomeutil.sourceforge.net/- Broad Institute) and contaminants such as chloroplast and eukaryotic DNA using BLAST [44]. The remaining set of high-quality 16S rRNA sequences were assigned to specific samples using multiplex barcodes incorporated during PCR amplification.

### Taxonomic assignment and OTU analysis

Each read was assigned a putative taxonomic identity using the RDP Bayesian classifier [45] (minimum confidence of 80%) as well as a secondary assignment using BLAST against the Greengenes database by using an *E *value cutoff of 1e-10 and the Hugenholtz taxonomy [46]. To describe the species-level structure of each microbial community, all sequences were clustered into operational taxonomic units (OTUs) using modules from the software package Mothur created by Pat Schloss [30]. Specifically, unique reads were aligned to the core Greengenes 16S template alignment using NAST [46]. Evolutionary distances were computed between all pairs of aligned sequences, which served as input to a furthest-neighbor clustering algorithm utilizing a distance threshold of 0.05 (i.e. 95% similarity). Good's coverage estimator [47] was computed for each sample using Mothur, which uses the following formula:

where Good's coverage of the *i*th sample (*C*_*i*_) depends on the total number of sequences in the sample (*N*_*i*_) and the number of singleton OTUs within that sample, *n*_*1i*_.

Statistical comparisons between environments were made using Metastats [28] (with 1000 permutations) to detect differentially abundant taxonomic groups at the phylum, class, genus, and OTU levels. Unless explicitly stated in the text, we employed a p-value significance threshold of 0.05.

### Enterobacteriaceae analysis

To perform a species-level analysis of the Enterobacteriaceae family, we created a database of 8,088 annotated 16S rRNA gene sequences from several Enterobacteriaceae species using the RDP database [48]. This database includes 451 16S rRNA sequences from *Salmonella *species, 951 from *E. coli *or *Shigella*, 762 from *Enterobacter*, 725 from *Pantoea*, and various other associated genera and environmental candidates.

We then searched all sequences from our samples against this database using BLASTN with default parameters and isolated any reads matching one of the reference genes with ≥ 98% identity along ≥ 95% of its length. NAST was then used to create a multiple sequence alignment of all matching reads and a reference set of 68 Enterobacteriaceae species that spanned *Salmonella*, *E. coli*, *Klebsiella*, *Pantoea*, *Enterobacter*, *Cronobacter*, and *Citrobacter*. The resulting MSA was trimmed by removing columns in the alignment with a high percentage of gaps (> 20%). The trimmed MSA was imported into Arb to create a neighbor-joining phylogenetic tree, using *Staphylococcus aureus *as an outgroup.

### Comparing alternative methodologies

To investigate the sensitivity of our major results to our particular methodology, we ran two alternate analyses employed by the CloVR virtual machine software package (http://clovr.org - Institute for Genome Sciences - University of Maryland Baltimore). These methodologies run similar analyses using Mothur [30] and Qiime [31] on a distributed cloud-computing architecture such as Amazon EC2. The high-quality dataset created after screening for contaminant and chimeras was used as input to the CloVR-16S pipeline.

## Abbreviations

*wg*: groundwater; *ws*: surface water; *pg*: groundwater-sprayed phyllosphere; *ps*: surface water-sprayed phyllosphere

## Authors' contributions

AT: conceived of the study, participated in its design and coordination, carried out field work and molecular biology experiments and drafted the manuscript, JRW: performed bioinformatics analyses and drafted the manuscript, DMP: participated in the study's design and coordination, carried out field and laboratory work and edited the manuscript, ARO: conceived of the study and edited the manuscript, CSW: conceived of the study, edited the manuscript and received the majority of funding needed to complete the research. All authors read and approved the final manuscript.

## Supplementary Material

Additional file 1 **Table S1: Bacterial classes abundance in tomato fruit surface and water samples**. Average relative abundance of sequences assigned to that class (mean), standard error of the corresponding average (SE) and p-value for the comparison between environments. Click here for file

Additional file 2 **Table S2: Bacterial genera abundance in tomato fruit surface and water samples**. Average relative abundance of sequences assigned to that genus (mean), standard error of the corresponding average (SE) and p-value for the comparison between environments. Click here for file
